# Comparison of the Efficacy of Continuous Saline Infusion to Prevent Catheter Occlusion: A Systematic Review and Meta-Analysis

**DOI:** 10.7759/cureus.92631

**Published:** 2025-09-18

**Authors:** Shunsuke Kondo, Yusuke Hirao, Shunsuke Yasuo, Yasushi Tsujimoto

**Affiliations:** 1 Internal Medicine, University of Hawaii, John A. Burns School of Medicine, Honolulu, USA; 2 Emergency Medicine, Kyoto Katsura Hospital, Kyoto, JPN; 3 Healthcare Epidemiology, Graduate School of Medicine and Public Health, Kyoto University, Kyoto, JPN

**Keywords:** catheter occlusion, catheter-related bloodstream infection, central venous catheter, continuous saline infusion, systematic review

## Abstract

Central venous catheters (CVCs) are essential in managing critically ill patients but are frequently complicated by occlusion, which can necessitate catheter replacement and increase the risk of complications such as catheter-related bloodstream infection (CRBSI). Continuous saline infusion devices have emerged as a potential intervention to maintain catheter patency, as continuous fluid administration is the common measure of fluid administration, though a persistently high occlusion rate remains in the high-risk population. This systematic review and meta-analysis assessed the efficacy of continuous saline infusion versus standard of care, including intermittent flushing and catheter lock, in preventing catheter occlusion. We searched MEDLINE, Embase, Cochrane CENTRAL, ClinicalTrials.gov, and the WHO International Clinical Trials Registry Platform (ICTRP) up to February 11th, 2025. Two randomized controlled trials (RCTs) involving 297 participants met the inclusion criteria. Both studies enrolled patients with newly placed CVCs, including critically ill populations, with an occlusion incidence of 33%. The included studies had an overall high risk of bias. Meta-analysis demonstrated a risk ratio (RR) of 0.51 (95% CI), corresponding to an absolute risk reduction of 267 occlusion events per 1,000 patients with low certainty. The incidence of CRBSI was not significantly different between groups (RR: 3.02, 95% CI: 0.12-73.52; low certainty). No catheter-related thrombosis or unexpected catheter removals were reported, with very low certainty of evidence. Adverse events were infrequent and primarily involved leakage at the catheter site. In conclusion, our findings suggest that continuous saline infusion may be considered for high-risk patients to prevent CVC occlusion. Further high-quality trials are needed to confirm these findings and evaluate their impact on clinically relevant outcomes such as thrombosis and infection.

## Introduction and background

Central venous catheters (CVCs) are widely used, especially for critically ill or perioperative patients [1]. They help measure hemodynamic variables that non-invasive methods cannot accurately assess and allow the safe delivery of blood products, medications, and nutrition when peripheral vein catheters are unsuitable. In the critically ill setting, CVCs are the route of vasopressors or nutrition, which cannot be delivered via a peripheral vein catheter [2]. Another common use of CVCs is for hemodialysis vascular access. In the ICU setting, bolus fluid administration is the common measure to deliver fluid to patients with CVC [3]. Even in the sepsis population, bolus fluid administration is mainly used to give IV fluid, as around 8.6% receive bolus fluid administration [4].

CVC occlusion is a frequent problem that often requires catheter removal or exchange. It increases the risk of CVC-related complications, such as catheter-related bloodstream infections (CRBSIs) and increased healthcare costs [5,6]. Current guidelines recommend intermittent normal saline flushing to prevent catheter occlusion, as multiple systematic reviews and meta-analyses, including recent Cochrane reviews and large randomized trials, demonstrated that intermittent heparin flushing has similar efficacy with no significant rate of catheter occlusion [7-9]. Normal saline intermittent flushing is generally recommended due to its lower cost and avoidance of heparin-associated risks, such as heparin-induced thrombocytopenia [10]. The National Kidney Foundation Kidney Disease Outcomes Quality Initiative (KDOQI) and the American Society of Clinical Oncology both recommend routine saline flushing as standard practice [11,12]. Despite the effort, a previous study reported that CVCs experience occlusion rates of approximately 4.95% within one week [13]. Catheter occlusion happens more frequently in ICUs because of the severity of the patients, non-standardized care, and the more frequent urgent catheter placement compared to the general wards, and the occlusion rate goes up to 33% in 10 weeks [14]. Also, catheter-related thrombosis is a more frequently reported complication, with one study noting a prevalence of 56% in ICU patients and approximately 14% to 18% in the general population [15,16].

Recently, continuous infusion devices have been developed as a novel approach to maintain catheter patency and prevent occlusion. These devices, utilizing continuous infusion technology, are used to deliver flushing fluid into the CVCs at a precise and steady rate of 2 mL per hour [17]. While a recent study demonstrated that heparin locking may be more effective than 0.9% sodium chloride locking for preventing occlusion in CVCs [18], evidence remains limited regarding optimal strategies for maintaining catheter patency and preventing occlusion [19]. In this context, continuous infusion devices have attracted attention, and several randomized controlled trials have evaluated their effectiveness, and both studies showed favorable outcomes [17,20]. However, no systematic review or meta-analysis has evaluated the efficacy of continuous infusion for catheter maintenance. We conducted a systematic review to evaluate the effectiveness of continuous infusion to prevent catheter occlusion.

## Review

Methods

We followed a pre-specified protocol [21]. The difference between the protocol and this review is provided in Supplement 1. We adhered to the Cochrane handbook [22], Grading of Recommendations Assessment, Development, and Evaluation (GRADE) informative statement [23], and Preferred Reporting Items for Systematic Reviews and Meta-Analyses (PRISMA) [24] when preparing this protocol and reporting the present study.

Eligibility Criteria

Types of studies: We included randomized controlled trials (RCTs), randomized crossover trials, and cluster RCTs that assess the efficacy of continuous flushing as compared to manual injection of saline. We did not apply language or country restrictions. We included all papers, including published, unpublished articles, abstracts of conferences [23], and letters.

We excluded non-randomized studies of interventions, such as quasi-experimental studies or observational studies. We did not exclude studies based on the observation period or publication year.

Study participants: Adult patients older than 16 years with a CVC, temporary hemodialysis catheter, or a peripheral insertion central catheter placement within 24 hours, regardless of the insertion site, were included.

The exclusion criteria were patients who must use alternative fluids for catheter locking; patients who need to use drugs that are incompatible with normal saline, such as lorazepam; patients who need to use drugs that cannot tolerate the cessation of infusion for catheter flushing. Patients receiving 24 hours of continuous infusion of the medications who do not require a lock to prevent obstruction were also excluded.

Intervention and Control

The intervention group used continuous normal saline infusion with a pre-filled elastic bag or syringe pump, which delivers fluid at a constant rate over 24 hours. The control group received standard care, which includes manual pulsatile normal saline flush or heparin solution flush at least every 24 hours with positive pressure lock or citrate lock. If medication is given through that catheter, the catheter is locked with positive pressure normal saline or heparin solution at the end of the infusion without routine additional positive pressure lock every 24 hours.

Outcomes

The primary outcomes of interest were catheter occlusion, which is defined by the Catheter Injection and Aspiration (CINAS) classification [25], incidence rate of CRBSI, and catheter-related thrombosis. Secondary outcomes of this study were CVC removal without a pre-specified plan and all adverse events.

Search Strategy

We searched the following databases: MEDLINE (PubMed), the Cochrane Central Register of Controlled Trials (Cochrane Library), and Embase (Dialog). The search strategies are listed in Appendix B. We also searched the US National Institutes of Health Ongoing Trials Register (ClinicalTrials.gov) and the WHO International Clinical Trials Registry Platform (ICTRP), listed in Appendix B. Also, we checked the reference lists of studies, including the reference lists of eligible studies and articles citing eligible studies. The search was conducted in February 2025. We asked the authors of original studies for unpublished or additional data.

Data Collection and Analysis

Selection of the studies: Two reviewers (SK and YH) performed independent data extraction of the included studies using a standardized data collection form. Any disagreements were resolved by discussion, and if this failed, a third reviewer acted as an arbiter (SY).

Assessment of risks of bias in included studies: Two reviewers (SK and YH) evaluated the risk of bias independently using the Risk of Bias 2 tool [26]. Disagreements between the two reviewers were discussed, and if this failed, a third reviewer (SY) acted as an arbiter, if necessary.

Data synthesis: Meta-analysis was performed using Review Manager software (RevMan 5.4.1, Cochrane Collaboration, London, UK). We used a random-effects model for meta-analysis. We pooled the relative risk ratios (RRs) and the 95% confidence intervals (CIs) for the following binary variables: number of occlusions of the catheter. We pooled the mean differences and the 95% CIs for the following continuous variables: catheter occlusion rate. If several different scales have been used in the included studies, we pooled the effect estimates using standard mean differences (SMDs). We summarized adverse events based on the definition by the original article, but we did not perform a meta-analysis. We performed the intention-to-treat (ITT) analysis for all dichotomous data as much as possible. For continuous data, we did not impute missing data based on the recommendation by the Cochrane handbook [22]. We performed a meta-analysis of the available data in the original study. When original studies only report standard error or p-value, we calculated the standard deviation based on the method by Altman and Bland [27]. If we do not know these values when we contact the authors, the standard deviation was calculated by confidence interval and t-value based on the method mentioned in the Cochrane handbook [22], or a validated method [28,29].

We evaluated the statistical heterogeneity by visual inspection of the forest plots and calculating the I2 statistic (I2 values of 0% to 40%: might not be important; 30% to 60%: may represent moderate heterogeneity; 50% to 90%: may represent substantial heterogeneity; 75% to 100%: considerable heterogeneity). When there is substantial heterogeneity (I2 > 50%), we assessed the reason for the heterogeneity. Cochran's chi^2^ test (Q-test) was performed for the I2 statistic, and a p-value less than 0.10 was defined as statistically significant.

We searched the clinical trial registry system (ClinicalTrials.gov and ICTRP) and performed an extensive literature search for unpublished trials. To assess outcome reporting bias, we compared the outcomes defined in trial protocols with the outcomes reported in the publications.

Rating the certainty of evidence: Two reviewers (SK and YH) evaluated the certainty of evidence based on the GRADE approach [30]. Disagreements between the two reviewers were discussed, and if this failed, a third reviewer (SY) acted as an arbiter, if necessary.

A summary of findings table was made for the following outcomes based on the Cochrane handbook [22]: catheter occlusion, CRBSI, catheter-related thrombosis, and unexpected catheter removal.

Results

**Figure 1:**
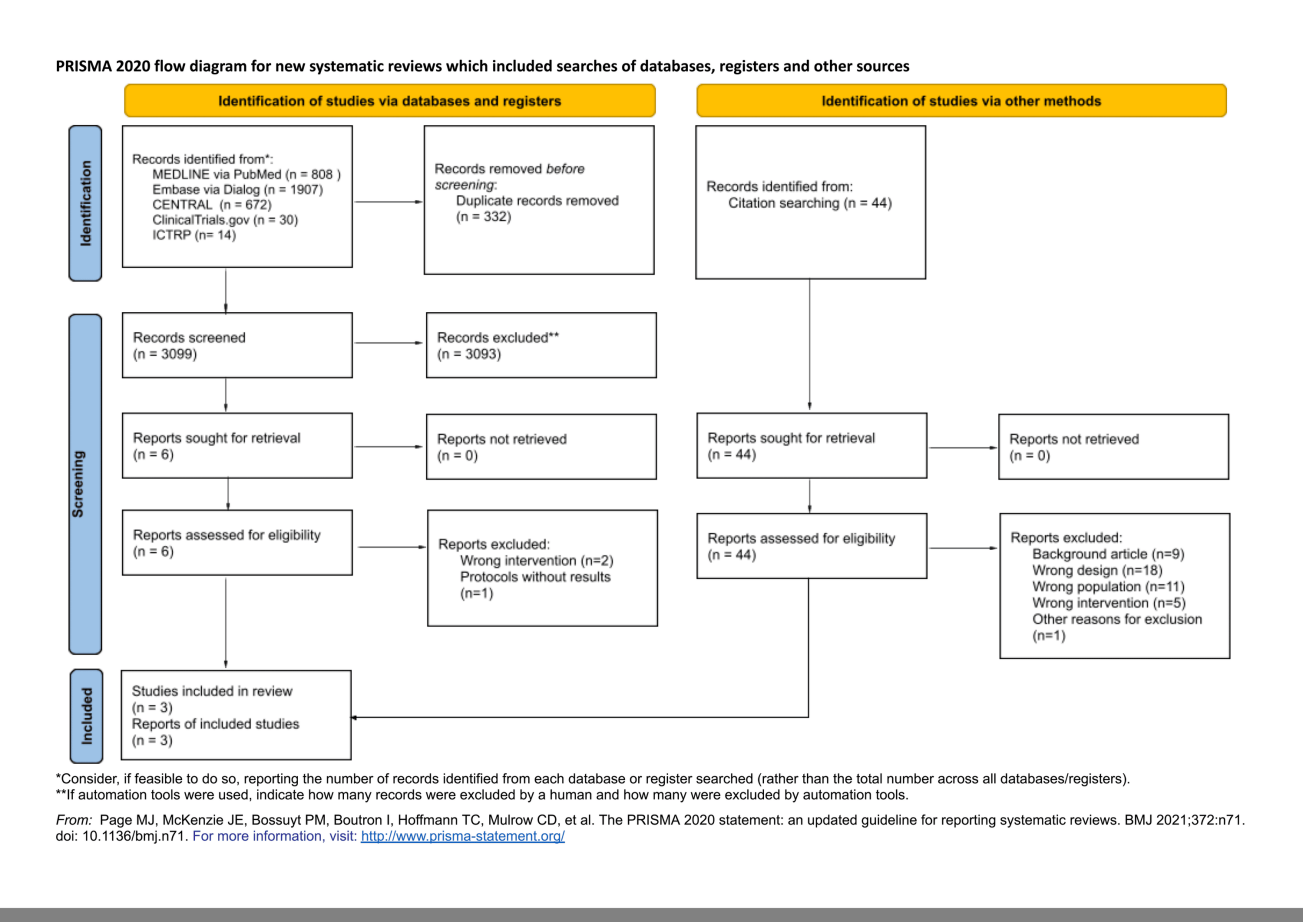
PRISMA (2020) flow diagram for a new systematic review, which included searches of databases, registers, and other sources. PRISMA: Preferred Reporting Items for Systematic Reviews and Meta-Analyses.

A total of 3,099 records were identified from the electronic database search, and 44 records were identified from the citation search. We retrieved full texts of six reports from the electronic database and 44 reports from citation searches for full assessment. Of these six full-text articles, we included three studies in our review, and one of them is an ongoing study, which is shown in Figure 1. The references of the excluded studies and the reasons for exclusion are summarized in Appendix C.

Table 1 shows the characteristics of the three included studies. All the included studies assessed the impact of the continuous normal saline infusion. Among the three studies, one study was not published, and the data were not available [31]. We found that the study participants were from the general population in one study [20], and one study included only critically ill patients [17]. Comparison of both studies was intermittent normal saline flushing. As only two studies were available, we performed a meta-analysis using these studies.

**Table 1:** Characteristics of included studies. RCTs: randomized controlled trials.

Study/country	N	Follow-up duration (days)	Population	Site of catheter (n)	Percent female	The main reason for ICU admission	Standard of care
RCTs							
Zhou et al. (2024) [20], China	251	7	Patients aged 14 to 80 years admitted to the hospital, who had a new centrally inserted central catheter placed within 24 hours, and the catheter was placed via the internal jugular or subclavian vein	Subclavian vein: 24. Internal jugular vein: 227	36.7%	Not reported	10 mL of saline was used to flush and positive pressure locking
Jia et al. (2024) [17], China	46	7	Patients aged 14 to 80 years admitted to ICUs who had a new centrally inserted central catheter placed within 24 hours, and the catheter was placed via the internal jugular or subclavian vein	Subclavian vein: 4. Internal jugular vein: 42	58.7%	Pneumonia = 10; abdominal pain = 6; acidosis = 5; pancreatitis/cholecystitis = 9; digestive tract perforation = 3; multiple site damage = 4; other reasons = 9	10 mL of saline was used to flush and positive pressure locking every 6 hours
Li et al. (2024) [31], China (ongoing)	Aim for 250	-	Patients aged 14 to 80 years admitted to ICUs who had a new centrally inserted central catheter placed within 24 hours, and the catheter was placed via the internal jugular or subclavian vein	-	-	-	10 mL of saline was used to flush and positive pressure locking every 6 hours

Primary Outcomes

**Figure 2:**
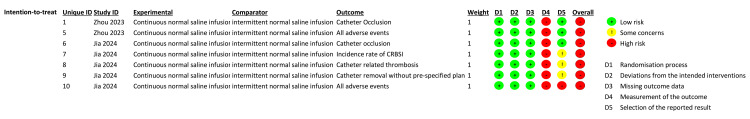
Summary of risk of bias. References: Jia et al. [17]; Zhou et al. [20].

We summarize the risk of bias in the outcomes of catheter occlusion rate and incidence rate of CRBSI in Figure 2. Both of the studies had an overall high risk of bias [17,20].

**Figure 3:**
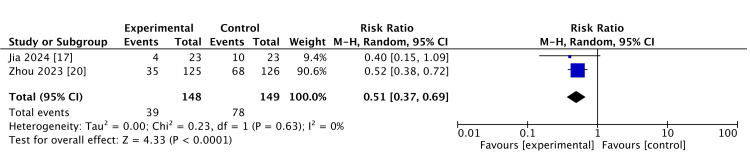
Catheter occlusion rate.

Catheter occlusion rate: Both studies were included in the meta-analysis. Continuous catheter flushing technique may reduce catheter occlusion rate (RR: 0.51, 95% CI: 0.37 to 0.69, I2 = 0%; two studies, 279 participants, low certainty of evidence). This is shown in Figure 3.

**Figure 4:**
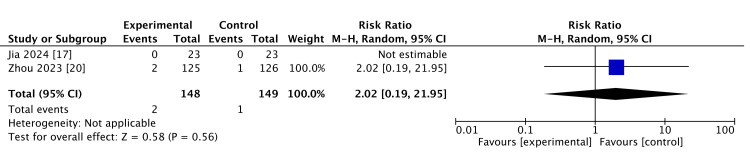
Incidence rate of catheter-related bloodstream infections (CRBSIs).

Incidence rate of CRBSI: Two studies reported CRBSI as an outcome measure. One study reported that there was no CRBSI in either the intervention or control groups. CRBSI may not be affected by continuous catheter flushing (RR: 3.02, 95% CI: 0.19 to 21.95; heterogenicity not applicable; two studies, 279 participants). This is shown in Figure 4.

Incidence rate of catheter-related thrombosis: Only one study reported this outcome. They reported no events in both intervention and control groups during the study period.

Secondary Outcomes

One study did not report catheter-related thrombosis and catheter removal without a pre-specified plan, although it was planned to measure in the protocol [20]. We sent emails twice to authors asking for the detailed outcome data of the incidence of catheter-related thrombosis and CVC removal without a pre-specified plan, but they did not respond, so no additional data are available. CVC removal without a pre-specified plan was reported in one study. They reported no events in both intervention and control groups during the study period. All adverse events were reported as qualitative synthesis and reported in the summary of findings table (Table 2 and Appendix A).

**Table 2:** Summary of findings: continuous catheter flushing technique compared to the intermittent manual injection of solution in adult patients who have had a new CVC inserted within the past 24 hours. * The risk in the intervention group (and its 95% CI) is based on the assumed risk in the comparison group and the relative effect of the intervention (and its 95% CI). GRADE Working Group grades of evidence High certainty: We are very confident that the true effect lies close to that of the estimated effect. Moderate certainty: We are moderately confident in the effect estimate: the true effect is likely to be close to the estimated effect, but there is a possibility that it is substantially different. Low certainty: Our confidence in the effect estimate is limited: the true effect may be substantially different from the estimated effect. Very low certainty: We have very little confidence in the effect estimate: the true effect is likely to be substantially different from the estimated effect. Patient or population: Adult patients who have had a new CVC inserted within the past 24 hours. Setting: Intervention: Continuous catheter flushing technique. Comparison: Intermittent manual injection of solution. CI: confidence interval; RR: risk ratio; RCTs: randomized controlled trials; CVC: central venous catheter; GRADE: Grading of Recommendations Assessment, Development, and Evaluation.

Outcomes	Anticipated absolute effects risk with the intermittent manual injection of the solution^*^	Anticipated absolute effects risk with the continuous catheter flushing technique^*^	Relative effect (95% CI)	No. of participants (studies)	Certainty of the evidence (GRADE)	Comments
Catheter occlusion rate	523 per 1,000	267 per 1,000 (194 to 361)	RR: 0.51 (0.37 to 0.69)	297 (2 RCTs)	⨁⨁◯◯ Low^a,b^	Continuous catheter flushing technique may reduce the catheter occlusion rate.
Catheter-related bloodstream infection (CRBSI)	0 per 1,000	10 per 1,000 (0 to 247)	RR: 3.02 (0.12 to 73.52)	297 (2 RCTs)	⨁⨁◯◯ Low^a,b^	CRBSI may not be affected by continuous catheter flushing.

Discussion

This systematic review found that the continuous catheter flushing technique may improve the catheter occlusion rate in patients who have CVCs placed within 24 hours compared to intermittent fluid administration, with low certainty of evidence. Our study also showed that the incidence rate of CRBSI may not be increased by using continuous catheter flushing. Only one study reported on the other clinically important outcomes, such as incidence of catheter-related thrombosis or catheter removal according to a pre-specified plan, but no events were observed. The main adverse event noted was leakage, and the incidence rate was low.

Continuous saline flushing demonstrates comparable effectiveness to other established interventions in preventing catheter occlusion. One meta-analysis reported that the intermittent heparin flushing was associated with a reduced risk of catheter occlusion by approximately 30-40% compared to intermittent saline flushing in patients with newly placed CVCs during the first 30 days [9]. Standardization of care for CVCs, which demonstrated improvement in occlusion rate in a previous systematic review [32], showed a 69% reduction without heterogenicity of the studies. Our study showed that catheter occlusion with the continuous flushing technique may reduce occlusion (RR: 0.51), though the overall number of catheter occlusions is lower in both intervention and comparison groups compared to the previous studies [15,16]. Also, CVCs made using new materials could prevent further occlusive events, though there is no available report focusing on the acute setting, as one report showed improvement in the long-term catheter placement [33]. Clinicians can apply continuous normal saline flushing with the standardized nursing education or new material CVCs, and this intervention may provide additional benefits in reducing the catheter occlusion rate. Patients who cannot receive heparin may also benefit from this intervention.

Although the possible downside of the continuous normal saline flushing is CRBSI, our study shows that it may not increase the rate of these complications. The rate of CRBSI is reported to be around 4.4% in the previous study [34], or 3.5 per 1000 catheter-days in high-income countries, as reported by the International Society for Infectious Diseases [35]. In contrast, we did not observe CRBSI in the control groups of the included study. We found more incidence of CRBSI in continuous normal saline flushing groups, but the absolute difference was modest.

Our review also highlights the need for future investigation to confirm the potential use of continuous saline infusion, as many of the outcomes in this review left uncertainty. The incidence rate of catheter-related thrombosis in our study was zero, whereas previous studies reported the incidence rate as 56% in ICU patients and 14% to 18% in the general population [15,16]. We therefore rated the certainty of evidence on this outcome as very low. CVC removal without a pre-specified plan was also not reported in one study and reported as zero in the other study. The adverse event rate in our study was higher compared to the previous studies, as 2-4 per 1000 insertions [5]. Studies included in our review counted minor adverse events such as leakage, which might increase the rate. One study with an expected sample size of 250 is ongoing, but larger RCTs measuring clearly defined important outcomes are needed to confirm our findings [31].

Our study has several limitations. First, we were able to find only two articles with results and one additional protocol. This device is new, and there are not many published studies yet. Second, we cannot perform the subgroup analysis or sensitivity analysis because the data are limited, and the author did not respond to our inquiry. Third, we could not obtain unpublished data on catheter-related thrombosis and catheter removal, though we had contacted the original investigators.

Despite these limitations, this study first summarized the efficacy of the continuous normal saline flushing compared to the intermittent catheter flushing technique.

Our study has several strengths. We followed the standard methods such as the Cochrane handbook and GRADE approach [22,30], and we did a comprehensive search, including unpublished protocols.

## Conclusions

In conclusion, clinicians may consider using continuous saline infusion devices for patients at high risk of catheter occlusion. However, our review also highlights several uncertainties that warrant attention when applying this intervention. Well-designed RCTs with larger sample sizes are needed to provide more definitive conclusions regarding the efficacy of this intervention.

## Appendices

Supplement 1: Difference between the protocol and review

We originally planned to include randomized crossover studies and cluster randomized trials, but there were no studies available. We also planned to perform subgroup analysis and sensitivity analysis, but we were not able to perform them because there were not enough data.

Appendix A

**Table 3:** Summary of findings and the results of narrative synthesis: continuous catheter flushing technique compared to the intermittent manual injection of the solution in adult patients who have had a new CVC inserted within the past 24 hours. Patient or population: Adult patients who have had a new CVC inserted within the past 24 hours. Setting: Intervention: Continuous catheter flushing technique. Comparison: Intermittent manual injection of the solution. * The risk in the intervention group (and its 95% CI) is based on the assumed risk in the comparison group and the relative effect of the intervention (and its 95% CI). CVC: central venous catheter; CRBSI: catheter-related bloodstream infection.

Outcomes	Anticipated absolute effects risk with the intermittent manual injection of the solution^*^	Anticipated absolute effects risk with the continuous catheter flushing technique^*^	No. of participants (studies)	Comments
Catheter-related thrombosis	See comments		46 (1 study)	One study reported that there were no catheter-related thromboses in both the intervention and control groups.
Unexpected catheter removal	See comments		46 (1 study)	One study reported that there were no catheter removals without any pre-specified plan, in both the intervention and control groups.
All adverse events	See comments		297 (2 studies)	The reported adverse events were as follows: one CRBSI in the intervention group, two leakages in the intervention group, and one leakage in the control group.

Appendix B

**Table 4:** Search strategy. ICTRP: International Clinical Trials Registry Platform.

Database	Search strategy
MEDLINE (PubMed) search strategy	Catheterization, Central Venous[MeSH Terms] Central Venous Catheters[MeSH Terms] 1 or 2 peripherally-inserted [tiab] PICC* [tiab] CVC*[tiab] CICC*[tiab] CVL*[tiab] PCVC*[tiab] central venous[tiab] or dialysis[tiab] OR jugular[tiab] OR femoral[tiab] OR subclavian[tiab] catheter*[tiab] 10 and 11 5 or 6 or 7 or 8 or 9 or 12 3 or 13 infusion pump[MeSH Terms] Infusions, Intravenous[MeSH Terms] 15 or 16 flushing[tiab] syringe pump*[tiab] elastic bag*[tiab] flow[tiab] infusion[tiab] administration[tiab] pump*[tiab] OR drip[tiab] OR drug*[tiab] infusion*[tiab] OR intravenous[tiab] 24 AND 25 17 OR 18 OR 19 OR 20 OR 21 OR 22 OR 23 OR 26 continuous[tiab] constant-rate[tiab] around-the-clock[tiab] sustained[tiab] steady-rate[tiab] uninterrupted[tiab] ongoing[tiab] maintenance[tiab] non-bolus[tiab] prolonged[tiab] constant flow delivery[tiab] 28 OR 29 OR 30 OR 31 OR 32 OR 33 OR 34 OR 34 OR 35 OR 36 OR 37 or 38 14 and 27 and 39 (((((((randomized controlled trial [pt]) OR (controlled clinical trial [pt])) OR (randomized [tiab])) OR (placebo [tiab])) OR (drug therapy [sh])) OR (randomly [tiab])) OR (trial [tiab])) OR (groups [tiab])) NOT (animals [mh] NOT humans [mh]) 40 and 41
CENTRAL (Cochrane Library) search strategy	[mh "Catheterization, Central Venous"] [mh "Central Venous Catheters"] 1 or 2 peripherally-inserted:ti,ab PICC*:ti,ab CVC*:ti,ab CICC*:ti,ab CVL*:ti,ab PCVC*:ti,ab "central venous":ti,ab OR dialysis:ti,ab OR jugular:ti,ab OR femoral:ti,ab OR subclavian:ti,ab catheter*:ti,ab 10 and 11 5 or 6 or 7 or 8 or 9 or 12 3 or 13 [mh "infusion pump"] [mh "infusion pump"] 15 or 16 flushing:ti,ab ("syringe" NEXT pump*):ti,ab ("elastic" NEXT bag*):ti,ab flow:ti,ab infusion:ti,ab administration:ti,ab pump*:ti,ab OR drip:ti,ab OR drug*:ti,ab infusion*:ti,ab OR intravenous:ti,ab 24 AND 25 17 OR 18 OR 19 OR 20 OR 21 OR 22 OR 23 OR 26 continuous:ti,ab constant-rate:ti,ab around-the-clock:ti,ab sustained:ti,ab steady-rate:ti,ab uninterrupted:ti,ab ongoing:ti,ab maintenance:ti,ab non-bolus:ti,ab prolonged:ti,ab "constant flow delivery":ti,ab 28 OR 29 OR 30 OR 31 OR 32 OR 33 OR 34 OR 34 OR 35 OR 36 OR 37 or 38 14 and 27 and 39
Embase (Dialog) search strategy	S1 EMB.EXACT.EXPLODE("randomized controlled trial") S2 EMB.EXACT.EXPLODE("controlled clinical trial") S3 TI(random*) OR AB(random*) S4 EMB.EXACT.EXACT("randomization") S5 EMB.EXACT.EXACT("intermethod comparison") S6 TI(placebo) OR AB(placebo) S7 TI(compare OR compared OR comparison) OR AB(compare OR compared OR comparison) S8 AB(evaluated OR evaluate OR evaluating OR assessed OR assess) AND AB(compare OR compared OR comparing OR comparison) S9 TI(open NEAR/1 label) OR AB(open NEAR/1 label) S10 (TI(double OR single OR doubly OR singly) NEAR/1 TI(blind OR blinded OR blindly)) OR (AB(double OR single OR doubly OR singly) NEAR/1 AB(blind OR blinded OR blindly)) S11 EMB.EXACT.EXACT("double blind procedure") S12 TI(parallel NEAR/1 group*) OR AB(parallel NEAR/1 group*) S13 TI(crossover OR "cross over") OR AB(crossover OR "cross over") S14 (TI(assign* OR match OR matched OR allocation) NEAR/6 TI(alternate OR group OR groups OR intervention OR interventions OR patient OR patients OR subject OR subjects OR participant OR participants)) OR (AB(assign* OR match OR matched OR allocation) NEAR/6 AB(alternate OR group OR groups OR intervention OR interventions OR patient OR patients OR subject OR subjects OR participant OR participants)) S15 TI(assigned OR allocated) OR AB(assigned OR allocated) S16 (TI(controlled) NEAR/8 TI(study OR design OR trial)) OR (AB(controlled) NEAR/8 AB(study OR design OR trial)) S17 TI(volunteer OR volunteers) OR AB(volunteer OR volunteers) S18 EMB.EXACT.EXACT("human experiment") S19 TI(trial) S20 S1 OR S2 OR S3 OR S4 OR S5 OR S6 OR S7 OR S8 OR S9 OR S10 OR S11 OR S12 OR S13 OR S14 OR S15 OR S16 OR S17 OR S18 OR S19 S21 (TI(random* NEAR/1 sampl* NEAR/8 ("cross section*" OR questionnaire* OR survey OR surveys OR database OR databases)) OR AB(random* NEAR/1 sampl* NEAR/8 ("cross section*" OR questionnaire* OR survey OR surveys OR database OR databases))) NOT (EMB.EXACT.EXPLODE("comparative study") OR EMB.EXACT.EXPLODE("controlled study") OR TI("randomised controlled") OR AB("randomised controlled") OR TI("randomized controlled") OR AB("randomized controlled") OR TI("randomly assigned") OR AB("randomly assigned")) S22 EMB.EXACT.EXACT("cross-sectional study") NOT (EMB.EXACT.EXPLODE("randomized controlled trial") OR EMB.EXACT.EXACT("controlled clinical study") OR EMB.EXACT.EXACT("controlled study") OR TI("randomised controlled") OR AB("randomised controlled") OR TI("randomized controlled") OR AB("randomized controlled") OR TI("control group") OR AB("control group") OR TI("control groups") OR AB("control groups")) S23 (TI("case control*") OR AB("case control*")) AND (TI(random*) OR AB(random*)) NOT (TI("randomised controlled") OR AB("randomised controlled") OR TI("randomized controlled") OR AB("randomized controlled")) S24 TI("systematic review") NOT TI(trial OR study) S25 (TI(nonrandom*) OR AB(nonrandom*)) NOT (TI(random*) OR AB(random*)) S26 TI("random field*") OR AB("random field*") S27 TI("random cluster" NEAR/4 sampl*) OR AB("random cluster" NEAR/4 sampl*) S28 (AB(review) AND TI(review)) NOT TI(trial) S29 AB("we searched") AND (TI(review) OR RTYPE(review)) S30 AB("update review") S31 AB(databases NEAR/5 searched) S32 ((TI(rat OR rats OR mouse OR mice OR swine OR porcine OR murine OR sheep OR lambs OR pigs OR piglets OR rabbit OR rabbits OR cat OR cats OR dog OR dogs OR cattle OR bovine OR monkey OR monkeys OR trout OR marmoset*) OR AB(rat OR rats OR mouse OR mice OR swine OR porcine OR murine OR sheep OR lambs OR pigs OR piglets OR rabbit OR rabbits OR cat OR cats OR dog OR dogs OR cattle OR bovine OR monkey OR monkeys OR trout OR marmoset*)) AND EMB.EXACT.EXPLODE("animal experiment")) S33 EMB.EXACT.EXPLODE("animal experiment") NOT (EMB.EXACT.EXPLODE("human experiment") OR EMB.EXACT.EXPLODE("human")) S34 S21 OR S22 OR S23 OR S24 OR S25 OR S26 OR S27 OR S28 OR S29 OR S30 OR S31 OR S32 OR S33 S35 S20 NOT S34 S36 EMB.EXACT.EXPLODE("central venous catheterization") S37 EMB.EXACT.EXPLODE("central venous catheter") S38 S36 OR S37 S39 TI(peripherally-inserted) OR AB(peripherally-inserted) S40 TI(PICC*) OR AB(PICC*) S41 TI(CVC*) OR AB(CVC*) S42 TI(CICC*) OR AB(CICC*) S43 TI(CVL*) OR AB(CVL*) S44 TI(PCVC*) OR AB(PCVC*) S45 TI(central venous) OR AB(central venous) OR TI(dialysis) OR AB(dialysis) OR TI(jugular) OR AB(jugular) OR TI(femoral) OR AB(femoral) OR TI(subclavian) OR AB(subclavian) S46 TI(catheter*) OR AB(catheter*) S47 S45 AND S46 S48 S39 OR S40 OR S41 OR S42 OR S43 OR S44 OR S47 S49 S38 OR S48 S50 EMB.EXACT.EXPLODE("infusion pump") S51 EMB.EXACT.EXPLODE("intravenous administration") S52 S50 OR S51 S53 TI(flushing) OR AB(flushing) S54 TI(syringe pump*) OR AB(syringe pump*) S55 TI(elastic bag*) OR AB(elastic bag*) S56 TI(flow) OR AB(flow) S57 TI(infusion) OR AB(infusion) S58 TI(administration) OR AB(administration) S59 TI(pump*) OR AB(pump*) OR TI(drip) OR AB(drip) OR TI(drug*) OR AB(drug*) S60 TI(infusion*) OR AB(infusion*) OR TI(intravenous) OR AB(intravenous) S61 S59 AND S60 S62 S53 OR S54 OR S55 OR S56 OR S57 OR S58 OR S61 S63 TI(continuous) OR AB(continuous) S64 TI(constant-rate) OR AB(constant-rate) S65 TI(around-the-clock) OR AB(around-the-clock) S66 TI(sustained) OR AB(sustained) S67 TI(steady-rate) OR AB(steady-rate) S68 TI(uninterrupted) OR AB(uninterrupted) S69 TI(ongoing) OR AB(ongoing) S70 TI(maintenance) OR AB(maintenance) S71 TI(non-bolus) OR AB(non-bolus) S72 TI(prolonged) OR AB(prolonged) S73 TI(constant flow delivery) OR AB(constant flow delivery) S74 S63 OR S64 OR S65 OR S66 OR S67 OR S68 OR S69 OR S70 OR S71 OR S72 OR S73 S75 S35 AND S52 AND S62 AND S74
ICTRP search strategy	(Central Venous Catheter* OR Central venous catheterization* OR peripherally-inserted OR PICC OR CVC OR CVL OR PCVC OR dialysis OR Jugular OR femoral OR subclavian) AND (infusion pump* OR Intravenous infusion OR flushing OR syringe pump* OR elastic bag* OR flow OR administration OR pump* OR drip OR drug*)
ClinicalTrials.gov search strategy	Condition or disease: central venous catheter OR catheterization OR catheterisation OR dialysis Intervention: continuous infusion OR infusion pump

Appendix C

**Table 5:** Excluded studies based on full screening.

No.	Reference	Reason
1	ChiCTR2200064007. A multi-center randomized controlled open-label clinical study on the nursing effect of different flushing and sealing techniques on central venous catheter (CVC). 2022	Protocol without outcome result
2	Ziyaeifard M, Alizadehasl A, Aghdaii N, Sadeghi A, Azarfarin R, Masoumi G, et al. Heparinized and saline solutions in the maintenance of arterial and central venous catheters after cardiac surgery. Anesth Pain Med 2015;5:e28056. https://doi.org/10.5812/aapm28056.	Wrong intervention
3	Abdelkefi A, Othman TB, Kammoun L, Chelli M, Romdhane N, Kriaa A, et al. Prevention of central venous line-related thrombosis by continuous infusion of low-dose unfractionated heparin, in patients with haemato-oncological disease. A randomized controlled trial. Thromb Haemost 2004;92:654‐661. https://doi.org/10.1160/th04-02-0087.	Wrong intervention
